# Effects of clonality on the genetic variability of rare, insular species: the case of *Ruta microcarpa* from the Canary Islands

**DOI:** 10.1002/ece3.571

**Published:** 2013-04-22

**Authors:** M Meloni, A Reid, J Caujapé-Castells, Á Marrero, J M Fernández-Palacios, R A Mesa-Coelo, E Conti

**Affiliations:** 1 Institute of Systematic Botany, University of ZurichZollikerstrase 107, Zurich, 8008, Switzerland; 2 Department of Environmental Systems Science, ETH ZurichUniversitätstrasse 6, Zurich, 8006, Switzerland; 3 Department of Molecular Biodiversity and DNA bank, Jardín Botánico Canario “Viera y Clavijo”- Unidad Asociada CSIC Apde Correos 14 de Tafira Alta, Las Palmas de Gran Canaria, 35017, Spain; 4 Island Ecology and Biogeography Research Group, Universidad de La LagunaTenerife, Spain; 5 GESPLAN – Gobierno de CanariasAvda. Tres de Mayo 71, Tenerife, 38005, Spain

**Keywords:** Clonal reproduction, genetic diversity, insular, microsatellite, rare, *Ruta microcarpa*

## Abstract

Many plant species combine sexual and clonal reproduction. Clonal propagation has ecological costs mainly related to inbreeding depression and pollen discounting; at the same time, species able to reproduce clonally have ecological and evolutionary advantages being able to persist when conditions are not favorable for sexual reproduction. The presence of clonality has profound consequences on the genetic structure of populations, especially when it represents the predominant reproductive strategy in a population. Theoretical studies suggest that high rate of clonal propagation should increase the effective number of alleles and heterozygosity in a population, while an opposite effect is expected on genetic differentiation among populations and on genotypic diversity. In this study, we ask how clonal propagation affects the genetic diversity of rare insular species, which are often characterized by low levels of genetic diversity, hence at risk of extinction. We used eight polymorphic microsatellite markers to study the genetic structure of the critically endangered insular endemic *Ruta microcarpa*. We found that clonality appears to positively affect the genetic diversity of *R. microcarpa* by increasing allelic diversity, polymorphism, and heterozygosity. Moreover, clonal propagation seems to be a more successful reproductive strategy in small, isolated population subjected to environmental stress. Our results suggest that clonal propagation may benefit rare species. However, the advantage of clonal growth may be only short-lived for prolonged clonal growth could ultimately lead to monoclonal populations. Some degree of sexual reproduction may be needed in a predominantly clonal species to ensure long-term viability.

## Introduction

Biodiversity on islands has intrigued biologists since Darwin (1859). One of the main reasons for the biological interest on islands lies in the fact that they represent “hotspots” of biodiversity, harboring species found nowhere else on earth (Myers et al. 2000; Whittaker and Fernàndez-Palacios 2007). The majority of insular species are rare and/or endangered (Frankham 1997, 1998; Ouborg et al. 2006). Population genetics theory attributes the high susceptibility to extinction of insular species to their small population size and isolation, which make them more prone to the effects of stochastic factors related to demographic variation, environmental fluctuations, and genetic drift (Carrol and Fox 2008). In particular, the low levels of genetic diversity that are thought to characterize insular endemic species limit their ability to adapt to a changing environment, making them more prone to extinction (Frankham 1998). Therefore, the genetic diversity of endemic species has important implications for their conservation.

Several factors, including demographic history, gene dispersal, and breeding system, influence patterns of neutral genetic diversity within populations and genetic differentiation among populations. In particular, selfing rate and the ability to propagate vegetatively have profound consequences for the genetic diversity of species (Hamrick and Godt 1989, 1996). Vegetative propagation leads to a clonal structure in which one clone (genet) may consist of several individuals (ramets). The most obvious genetic signature of vegetative propagation in a population is the presence of repeated multilocus genotypes (MLGs) and, as a consequence, the nonrandom association of alleles at different loci (linkage disequilibrium, LD). It was often assumed that clonal organisms harbor low levels of genetic diversity. However, this assumption was usually a by-product of using genetic markers with low power of resolution (Arnaud-Haond et al. 2005). Different extents of clonality will have varying consequences on the genetic structure of populations affected by vegetative propagation. Mixed clonal/sexual reproduction seems to have negligible genetic effects if the proportion of vegetative propagation is low, while high rates of clonality affect most genetic indexes (Balloux et al. 2003).

Heterozygosity and allelic diversity at each locus are expected to increase under clonal propagation (Birky 1996; Balloux et al. 2003). In strictly clonal organisms, in fact, the alleles at one locus evolve independently and accumulate different mutations over time (Butlin 2000; Halkett et al. 2005). The accumulation of mutations in absence of sex promotes the divergence between alleles at a single locus within individuals, a phenomenon known as “Meselson effect” (Balloux et al. 2003). While high levels of clonality tend to increase genetic variation within populations, an opposite effect is expected on genetic differentiation among populations and on genotypic diversity, both decreasing with the rate of clonal reproduction (Balloux et al. 2003; Halkett et al. 2005). In this study, we investigate the genetic consequences of clonality in a rare, insular species.

*Ruta microcarpa* Svent (Rutaceae) is a narrow endemic of the Canary Islands listed as critically endangered (CR) under the Spanish red list of vascular flora (Bañares et al. 2004; Moreno 2008). Its distribution is restricted to the North of the island of La Gomera, where it is present with a total of approximately 250 individuals forming three main populations (60–130 individuals) and some very small populations (up to five individuals each; Bañares et al. 2004; Moreno 2008). Field observations highlight the scarcity of seedlings for this species and the occurrence of vegetative propagation by rhizomes and stolons (Bañares et al. 2004). Given the endemic character of *R. microcarpa,* its conservation status and the occurrence of vegetative propagation, this species represents an ideal case study to determine the effects of clonality on rare and endangered species, especially on islands. The main goals of this study are: (i) to genetically check for the occurrence of vegetative reproduction (i.e., clonality) in *R. microcarpa* populations; (ii) to assess the amount and distribution of genetic diversity using highly polymorphic markers (microsatellites) and (iii) to determine the effects of clonal propagation on genetic diversity in this species.

## Materials and Methods

### Study organism

*Ruta microcarpa* Svent (Rutaceae), a shrub up to 0.80–1.5 m, is characterized by dense branches, remotely toothed leaves, and relatively small fruits (Sventenius 1969; Bramwell and Bramwell 1994; Bañares et al. 2004). The small, yellowish, tetramerous flowers are hermaphroditic and pollination is favored by Diptera and Hymenoptera, while dispersal is thought to be effected mainly by birds and lizards (Bañares et al. 2004; M. Nogales, pers. comm.). It blooms from March to May, fruiting in May–June. The habitat is mostly hilly, open areas or steep rocky slopes, including screes, although some populations have colonized abandoned cultivation areas along with other xeric species, for example *Euphorbia obtusifolia*. While ploidy level analyses exist for most members of the genus *Ruta*, there is currently no information for *R. microcarpa*. In this regard, it should be noted that *R. microcarpa* is included in a clade with two other endemic species of the Canary Islands, *R. oreojasme* and *R. pinnata*, which are tetraploid, as is their mainland sister species, *R. montana* (Stace et al. 1993; Salvo et al. 2010), thus it is likely that the species under examination is also a tetraploid.

### Sample collection

**Figure 1:**
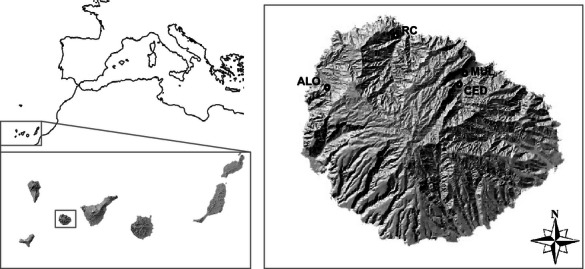
Sampling localities of the studied populations of *R. microcarpa*. Information on each population is provided in Table 1

Analyses were conducted on a total of 73 individuals from four wild populations of *R. microcarpa* (Fig 1), which represented the three largest populations known in La Gomera (Mulagua, MUL; Alojera, ALO; and Roque Cano, RC) and one smaller population (Camino del Cedro, CED). Populations MUL and RC showed discontinuities in their distributions. MUL was crossed by a road that separated subpopulation MUL1 on a steep slope below the road and subpopulation MUL2 on a gentler slope above the road. Two groups of plants were quite distinctly separated in the space of RC, even though close to each other (250–300 m): RC1 located in a small area (20 × 20 m) in an escarpment subjected to landslides, RC2 occupying a bigger (200 × 50 m), undisturbed area. Since clonal reproduction is thought to occur in this species (Bañares et al. 2004), samples were collected sufficiently far from each other (>10 m) to reduce the probability of sampling ramets from the same genet. Leaf tissue samples were collected in March–June of 2010 and 2011 and were preserved in silica gel.

### DNA extraction

Total genomic DNA was extracted using the QIAGEN® DNeasy plant mini kit (QIAGEN, Hombrechtikon, Switzerland), following the manufacture's guidelines. Since the plants generated very viscous cell lysate, minor modifications were applied to the protocol to optimize genomic DNA quality and yield. Specifically, we increased the volume of buffer AP1 (from 400 μL to 600 μL), buffer AP2 (from 130 μL to 200 μL), and RNase A (from 4 μL to 6 μL) and applied a longer incubation time (to 15 min) with buffer AP1 for cell lysis. Genomic DNA quality and quantity were checked by gel electrophoresis and using a NanoDrop spectrophotometer.

### Microsatellite amplification and genotyping

After screening 10 microsatellites (SSR, simple sequence repeat) newly developed for *R. oreojasme* (Meloni et al. 2013), nine loci were found to amplify reliably in all individuals, hence were used to genotype all 73 *R. microcarpa* individuals. Information on the selected SSRs is summarized in Table S1.

Polymerase chain reaction (PCR) amplifications were performed following the method described by Schuelke (2000). PCR was performed in 25 μL containing approximately 20 ng of genomic DNA, 2.5 μL of 10× reaction buffer, 0.5 μL of each dNTP (10 m004D), 1 μL of MgCl_2_ (50 mmol/L), 0.2 μL of the forward primer (10 μmol/L), 0.5 μL of the reverse primer (10 μmol/L), 0.5 μL of the fluorescently labelled M13(-21) primer (FAM, NED, VIC, PET; 10 μmol/L), and 0.1 μL of Taq DNA polymerase (5 U/μl; Bioline GmbH, Luckenwalde, Germany). An additional 1.0 μL of Bovine Serum Albumin (BSA, 20 mg/mL) was employed to increase the amplification success of the locus RO66. PCR was carried out using a T1 Thermocycler (Biometra GmbH, Goettingen, Germany) under the following conditions: initial denaturation at 94°C for 3 min, followed by 30 cycles of 94°C for 30 sec, T_a_ for 45 sec (see Table 1), and 72°C for 1 min. The incorporation of the fluorescently labelled M13(-21) primer occurred in the following eight cycles of 94°C for 30 sec, 53°C for 45 sec, and 72°C for 1 min, followed by a final extension step of 72°C for 5 min. Up to four PCR products of different primer sets with different fluorescent dyes (Table S1) were pooled for each individual and separated by capillary electrophoresis on an AB3130xl Genetic Analyzer (Applied Biosystems). Alleles were sized against the internal size standard GeneScan™ LIZ500™ (Applied Biosystems, Foster City, CA) and scored using GeneMapper® software Version 4.0 (Applied Biosystems).

**Table 1:** Description of *R. microcarpa* populations surveyed in this study (see also Fig. 1)

Population	Sub-Population	Location	Population size	Sample number	Coordinates	Altitude (m)	Area (km^2^)	Threat
ALO	–	Teguerguenche	63	19	N28° 08.840′ W17° 19.078′	633	2	Grazing competition
RC	RC1 RC2	Roque Cano	63	11 15	N28° 11.048′ W17° 15.265′ N28° 10.445′ W17° 15.633′	275 450	1	Competition Landslides Competition
MUL	MUL1 MUL2	Mulagua	130	10 15	N28° 08.576′ W17° 11.885′ N28° 08.385′ W17° 11.955′	471 478	1	Grazing Anthropogenic effect
CED	–	Camino del Cedro	4	4	N28° 08.867′ W17° 12.317′	400	–	–

### Statistical analysis

A maximum of two alleles per locus and per individual were detected in all populations. This may indicate that (1) the species is diploid or (2) the species is an extreme allotetraploid in which each chromosome exclusively pairs with its homolog, leading to disomic inheritance (Stift et al. 2008). Since in both cases genetic analyses can be performed with standard population genetic tools developed for diploid organisms (Stift et al. 2008), our analyses were conducted assuming a diploid status of *R. microcarpa*.

#### Existence and extent of clonal propagation

Multilocus genotypes (MLGs) were assigned manually. Samples with missing data were assigned to a MLG only when all other known MLGs could be excluded as possible genotypes. Samples differing by one or two alleles were re-genotyped to exclude scoring errors. Because individuals with the same MLG found in populations with both sexual and vegetative reproduction can be either ramets of the same genet or derive by chance from distinct events of sexual reproduction, we used the program GIMLET 1.3.2 (Valièr 2002) to estimate the probability that two individuals, randomly sampled from a population, share the same MLG by chance (probability of identity: PI); *PI*_unbiased_ and *PI*_sibs_ were measured to correct for small samples of individuals and for presence of shared ancestry among individuals, respectively.

After the occurrence of clonal propagation was confirmed in all populations, the extent of clonality was measured. In order to account for somatic mutations and to avoid underestimation of clonality, the program GenClone2 (Arnaud-Haond and Belkhir 2007) was used to construct a histogram of the frequency distribution of pairwise genetic distances based on a stepwise mutational model. The valley between the first two peaks of the histogram was used as a threshold: samples with pairwise genetic distances smaller than this threshold were assigned to the same clone (Meirmans and Tienderen 2004; Arnaud-Haond and Belkhir 2007; Rozenfeld et al. 2007; Zhang et al. 2010; see Table S2 for samples assignment). Three different genotypic diversity indexes were calculated. The first measure was *G/N*, that is, the ratio between the number of MLGs and the total number of individuals in a population (Halkett et al. 2005). A *G/N* ratio close to zero (all individuals share the same MLG) indicates strict clonality, while a *G/N* ratio equal to one (each individual has a distinct MLG) indicates sexual reproduction (Ivey and Richards 2001). The second measure was MLG diversity: 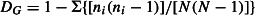, where *n*_*i*_ is the number of individuals with MLG *i* and *N* is the total number of individuals in a population (Pielou 1969). This index measures the probability that two individuals randomly selected from a population of *N* individuals will have different MLGs. A value of zero indicates that there is only one dominant clone, while *D*_G_ = 1 suggests that every individual has a different genotype. The third measure was Fager's genotypic evenness: *E* = *D*_G_/*D*_max_, where *D*_max_ = [*N*(*k*−1)]/[*k*(*N*−1)] and *k* is the number of genotypes in a population (Fager 1972). Evenness measures how genotypes are distributed within a population. Similar to the first two measures, evenness ranges from zero for a population in which all individuals have the same genotype to one when all genotypes in a population occur at the same frequency. The use of genotypic evenness allows for the comparison of populations with different numbers of clones (Montalvo et al. 1997; Arnaud-Haond et al. 2005). Analyses on clonality were conducted considering subpopulations MUL1, MUL2, RC1, and RC2 as separate entities (for a total of six populations).

#### Amount and distribution of genetic variability

Population genetic analyses were based on a ‘corrected’ dataset in which all individuals with the same MLG were considered as ramets of a single genet (for a total of 17 individuals, one per MLG). Individuals differing by few somatic mutations were considered different genets. This choice was motivated by the fact that in plants, in which germ cells differentiate from somatic tissues, somatic mutations have a great probability of being incorporated into gametes and passed on to the next generation (van Oppen et al. 2011). Somatic mutations, thus, represent an important source of heritable variation for clonal plants. Because the corrected dataset resulted in a population size of only one individual for RC1, both RC1 and RC2 were grouped together to form population RC. The total number of alleles, as well as observed (*H*_o_) and expected (*H*_e_) heterozygosity were calculated across loci for each population. Populations were tested for deviation from Hardy–Weinberg equilibrium using Fisher's exact test and the Markov chain algorithm (Guo and Thompson 1992). *F*-statistics were estimated following a standard Analysis of Molecular Variance (AMOVA), as described in Weir and Cockerham (1984). The fixation index, *F*_IS_, was estimated in order to assess the departure from Hardy–Weinberg expectations due to nonrandom mating. Pairwise comparisons of population differentiation were estimated using *F*_ST_. Genetic differentiation among populations was also estimated by *R*_ST_, an analogue of *F*_ST_ specific for microsatellite data, employing a stepwise mutation model (SMM, Slatkin 1995). Because indexes that take into account the SMM are affected by high variance when a small number of loci (<20) is used and/or populations are small (<10; Gaggiotti et al. 1999), we consider *F*_ST_ more suitable than *R*_ST_ to estimate genetic differentiation among populations and all related genetic indexes. In order to assess the hierarchical distribution of genetic variation, an AMOVA was conducted following the procedure of Excoffier et al. (1992) and using 999 random permutations of the data. Linkage disequilibrium between all different pairs of loci was tested at the single population level and across all populations using Fisher's exact test. To check for isolation by distance, a Mantel test (Mantel 1967) was applied to the matrices of pairwise population differentiation (calculated as *F*_ST_/(1−*F*_ST_)), and of log-transformed geographic distances between populations with 1000 random permutations. In order to determine the effect of genetic drift and gene flow on population structure, a scatter plot of pairwise genetic (*F*_ST_) and geographic distances was evaluated (Hutchison and Templeton 1999). The number of reproductively successful migrants per generation (*N*m), based on *F*_ST_ values, was estimated to indirectly measure gene flow. The software packages used for population genetic analyses were GENEPOP 4.0 (Rousset 2008) and GenAlEx v.6.41 (Peakall and Smouse 2001).

## Results

### Presence and extent of clonal propagation

In the six analyzed populations of *R. microcarpa*, we found a total of 17 different MLGs. After correcting for somatic mutations, 14 clones were considered: six in population ALO, three in population MUL2, two in population CED, and only one in each of populations RC1, RC2, and MUL1 (Table S2). No MLGs were found in common between populations. All populations were affected by clonality: the joint probability that individuals with the same MLG occurred by chance was significantly low (*PI*_unbiased_=1.086E-08; *PI*_sibs_=8.626E-04); therefore, it is highly likely that individuals sharing the same MLG are ramets of the same genet.

The population with the lowest *G/N* ratio was RC2 (0.067), the highest value being found in CED (0.500; Table 2). The results did not change after considering MUL and RC as single populations with no subdivision. Multilocus genotype diversity (*D*_G_) ranged from zero (RC1, RC2, MUL1) to 0.562 (ALO), with a mean value of 0.215 (Table 2). Genotypic evenness (*E*) ranged from 0.637 (ALO) to 0.750 (CED; Table 2). It was not possible to calculate the index *E* for RC1, RC2, and MUL1 since *D*_G_ reached the lowest value of 0.000 for these populations.

**Table 2:** Measures of genotypic diversity: ratio between the number of multilocus genotypes and the total number of individuals (*G/N*); multilocus genotype diversity (*D*_G_); genotypic evenness (*E*). For abbreviations of populations and subpopulations see Table 1

Population	*G/N*	*D*_G_	*E*
ALO	0.333	0.562	0.637
RC1	0.091	0.000	–
RC2	0.067	0.000	–
MUL1	0.100	0.000	–
MUL2	0.200	0.514	0.724
CED	0.500	0.500	0.750
Mean	0.215	0.263	

### Amount and distribution of genetic variability

#### Genetic diversity

A total of 52 distinct alleles were identified. With the exception of RO72, all loci were polymorphic, with the number of alleles identified at each locus across all populations ranging from three to ten (Table S1). Private alleles were found in each population: one in RC1; two in RC2, MUL1 and MUL2; eight in CED and nine in ALO. Since RO72 was monomorphic in all populations of *R. microcarpa*, it was excluded from further analyses. Based on the departure of *F*_IS_ from zero, most of the populations were at Hardy–Weinberg equilibrium across loci (*P* > 0.05). The only exception was ALO, for which only one locus was found at equilibrium (RO79; *P* = 0.935). Gene diversity, inferred from Nei's heterozygosity (*H*_e_), was homogeneously distributed across populations and relatively low, ranging from 0.375 in MUL1 to 0.552 in RC. Total gene diversity within the species was *H*_e_=0.410. *H*_o_ always showed values higher than *H*_e_ (*F*_IS_ values were always negative; Table 3), meaning that the departure from Hardy–Weinberg expected genotype frequencies was always associated with an excess of heterozygotes.

**Table 3:** Genetic variability within *R. microcarpa* populations. Abbreviations: *A* number of alleles, *H*_o_ observed heterozygosity, *H*_e_ expected heterozygosity, *F*_IS_ fixation index; SD, standard deviation. For abbreviations of populations and subpopulations see Table 1

Population	*A*	*H*_o_ ± SD	*H*_e_ ± SD	*F*_IS_
ALO	20	0.797 ± 0.138	0.474 ± 0.077	−0.680
RC	14 (RC1)/16 (RC2)	0.833 ± 0.126	0.552 ± 0.078	−0.509
MUL1	13	0.500 ± 0.189	0.375 ± 0.091	−0.333
MUL2	18	0.500 ± 0.126	0.448 ± 0.075	−0.116
CED	17	0.625 ± 0.157	0.469 ± 0.093	−0.333
Overall		0.651 ± 0.067	0.410 ± 0.037	−0.578

#### Linkage disequilibrium

Genotypic linkage disequilibrium was analyzed for each pair of loci for each population and across all populations. For 131 out of 168 pairwise combinations of loci it was impossible to perform the test, because at least one of the loci was monomorphic in the analyzed population. No significant linkage disequilibrium at the 1% level was detected on all the pairs of loci for which the test was possible.

#### Genetic differentiation among populations

Genetic differentiation among populations was measured using both *F*_ST_ and *R*_ST_ (Table 4). Values were always statistically significant (*P* < 0.05). *F*_ST_ values were high, ranging between 0.285 (MUL2-CED) and 0.512 (MUL1-MUL2); *R*_ST_ values were higher and showed a less homogeneous pattern with some populations highly differentiated (ALO, MUL1; 0.711 < *R*_ST_ < 0.974) and other populations characterized by lower genetic differentiation (RC-MUL2, RC-CED, MUL2-CED; 0.269 < R_ST_ < 0.306). The overall genetic differentiation between populations was significant, with *F*_ST_ = 0.446 (*P* = 0.01) and *R*_ST_ = 0.869 (*P =* 0.01).

**Table 4:** Pairwise population estimates of *F*_ST_ (below diagonal) and *R*_ST_ (above diagonal). For abbreviations of populations and subpopulations see Table 1

	ALO	RC	MUL1	MUL2	CED
ALO	–	0.895***	0.974**	0.931***	0.859***
RC	0.399***	–	0.711**	0.269*	0.306***
MUL1	0.499**	0.421***	–	0.898***	0.775***
MUL2	0.442***	0.394***	0.512***	–	0.301*
CED	0.492***	0.466***	0.471*	0.285***	–

* *P* < 0.05;

** *P* < 0.01;

*** *P* < 0.001.

#### Isolation by distance and gene flow

**Figure 2:**
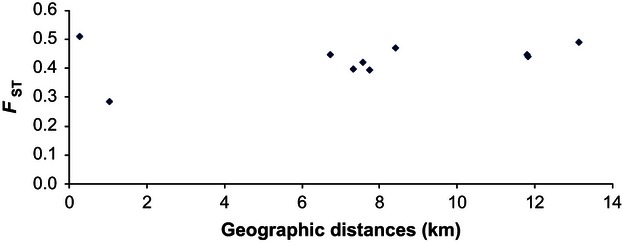
Scatter plot of *F*_ST_ estimates (Weir and Cockerham 1984) against geographic distances (km) calculated for each pairwise combination of populations.

No significant correlation between genetic differentiation (measured with *F*_ST_) and geographic distances among populations was shown by the Mantel test (*P* = 0.616, *R*^2^ = 0.043). The scatter plot of genetic and geographic distances separating each pairwise combination of populations (Fig 2) suggested that genetic structure has been more influenced by drift than gene flow. The number of migrants between populations (based on *F*_ST_) was very low (0.238 < *N*m < 0.628). Values ranged from 0.238 to 0.385 for all pairs of populations except CED-MUL2, for which the index was slightly higher (0.628). The total migration rate across populations was 0.127 individuals per generation.

#### AMOVA

The hierarchical distribution of genetic variation was estimated using an AMOVA and performed on two datasets: (A) with the six defined populations (ALO, MUL1, MUL2, RC1, RC2, CED), and (B) with the subpopulations of RC defined as a single population (see above). In both cases, the among-population element explained most of the total amount of variation: 82% and 62% for six and five populations, respectively.

## Discussion

### Genetic diversity, clonal propagation, and insularity

*Ruta microcarpa*, with its small, isolated populations, and phenotypic evidence of clonality, provides a distinctive model to study the effects of clonal reproduction on the genetic structure of rare island species. The population genetic results reported here show that clonality represents a common reproductive strategy for all analyzed populations and that it appears to counteract some of the effects of small population size and isolation by increasing heterozygosity, polymorphism, and allele richness in *R. microcarpa* populations.

Although the amount of genetic variability we found in *R*. *microcarpa* is low, it is higher than expected if considering the geographic restriction to a single island, the small population sizes, and the low total number of individuals in the species. According to population genetic theory, in fact, rare insular species should be characterized by overall low levels of gene diversity, a low number of alleles per locus, low polymorphism (i.e., several fixed loci), and a high rate of linkage disequilibrium among loci (Hamrick and Godt 1996; Frankham 1998; Frankham et al. 2002; Ouborg et al. 2006). The high number of heterozygotes detected in *R*. *microcarpa* (*H*_o_ = 0.651; Table 3) together with the relatively high levels of gene diversity (*H*_e_ = 0.410; Table 3) and the detection of just one monomorphic locus are unexpected results for rare insular species and may represent the genetic effects of the high allelic divergence driven by clonality (Halkett et al. 2005). Support for this interpretation comes from the observation that, contrary to our results in *R. microcarpa*, low values of genetic diversity were found for sexually reproducing Canarian endangered species (*H*_e_ = 0.2 for *Anagyris latifolia*, González-Pérez et al. 2009; *H*_o_ = 0.113, *H*_e_ = 0.306 for *Lotus kunkelii*, Oliva-Tejera et al. 2006; *H*_o_ = 0.100, *H*_e_ = 0.112 for *Cistus chinamadensis ssp. gomerae*, Batista et al. 2001), while values of genetic diversity were similar to those found in this study for other endangered clonal species such as the Canarian endemic *Sambucus palmensis* (*H*_o_ = 0.550, *H*_e_ = 0.499; Sosa et al. 2010) *and the Southern Appalachian endemic Spiraea virginiana* (*H*_o_ = 0.503, *H*_e_ = 0.391; Brzyski and Culley 2011).

As commonly detected in other plant species (Eckert et al. 2003; Travis et al. 2004; Tsyusko et al. 2005), we found that clonality does not equally affect the different populations of *R. microcarpa*. According to our data, RC1, RC2, and MUL1 are strictly clonal, while in populations ALO, MUL2, and CED sexual and asexual recruitment strategies seem to contribute equally to reproduction (Table 2). Two hypotheses may explain the pattern of strict clonality that we found in populations RC1, RC2, and MUL1: i) there is no sexual reproduction in these populations, for even few events of sexual reproduction per generation should be sufficient to prevent an extreme monoclonal genotypic pattern (Bengtssom 2003) and ii) no seedling recruitment occurred over a relatively long period of time. However, a few seedlings were observed during field sampling in RC1 and RC2 (À. Marrero, pers. comm.), suggesting that occasional events of sexual reproduction take place in these populations thus supporting the hypothesis of no seedling recruitment. Moreover, allelopathy has been observed for some *R. microcarpa* populations (R. M. Coelo, pers. comm.), further suggesting that some allelochemicals might inhibit seedlings growth in RC1, RC2 and MUL1.

Many plant species combine sexual and vegetative reproduction (Richards 1986). The balance between sex and clonal growth varies between and within species (Honnay and Bossuyt 2005) and is mainly driven by environmental fluctuations (including both episodic and continuous changes), making the two modes of reproduction successful under different circumstances (Honnay and Bossuyt 2005; Silvertown 2008). Vegetative propagation has ecological costs mainly related to the increased size of clonal plants, resulting in higher resource uptake, increased space occupied, higher probability to interact with other conspecific or heterospecific plants, reduced pollen dispersal, and increased geitonogamous self-pollination, all leading to fitness costs associated with inbreeding depression and pollen discounting (Bushakra et al., 1999; Honnay and Jacquemyn 2008; Vallejo-Marín et al. 2010). Despite the mentioned costs, species that can reproduce clonally have several potential ecological and evolutionary advantages: they can persist in habitats that may not be consistently favorable for sexual reproduction, can better uptake resources in heterogeneous environments, spread the risk of death among ramets, and can increase the attraction of pollinators by increasing floral display size (Honnay and Jacquemyn 2008; Vallejo-Marín et al. 2010).

In the case of *R. microcarpa*, clonality could provide advantages on two fronts: (1) in small, isolated populations clonal reproduction may provide a form of reproductive assurance by guaranteeing the survival of the species in case of limited pollinator service or absence of mates (Lhuillier et al. 2006; Silvertown 2008); (2) in harsh environments, including steep and windswept ridges or areas with rocky soil affected by frequent landslides, germination of seeds is unlikely, whereas new individuals can be easily generated through clonal propagation (Lhuillier et al. 2006). The combination of population size and type of habitat characterizing each population of *R. microcarpa* can explain the different levels of clonal propagation we found in different populations. A higher rate of asexual reproduction, in fact, is found in MUL1 (*D*_G_ = 0.200; Table 2) than in MUL2 (*D*_G_ = 0.514; Table 2), the former consisting of only a few individuals located on a cliff below a road, the latter comprising more individuals and located on a gentle slope in an open area. In population ALO (composed of many large individuals, located in an open area in the NW part of the island, and with no obvious human impact detected) we found the highest genotypic diversity. Lhuillier et al. (2006) found a similar pattern in *Santalum insulare*, where populations more subjected to overexploitation, environmental stress, and human impact showed higher levels of clonality. A higher incidence of clonal reproduction in populations threatened by human activities was also found in non insular species (Kenningtom and James 1997; Warburton et al. 2000; Smith et al. 2003).

The low values of genotypic diversity (*G/N* = 0.215, *D*_G_ = 0.263; Table 2) discovered in the analyzed populations of *R. microcarpa* confirm the high overall degree of clonality of this species, especially when compared with other species characterized by small, naturally isolated populations that occur on continents. Lower levels of clonality, for example, were inferred in the endangered species *Cypripedium calceolus* (*D*_G_ = 0.97; Brzosko et al. 2002), in the rare species *Allium triccocum* (*D*_G_ = 0.87; Vasseur 2001) and in threatened populations of *Eucalyptus curtisii* (*G/N* = 0.53, *D*_G_ = 0.72; Smith et al. 2003). Levels of clonality similar to those of *R. microcarpa* were retrieved in the endangered insular Pacific tree *Santalum insulare* (*G/N* = 0.35, *D*_G_ = 0.43; Lhuillier et al. 2006). The observation in *R. microcarpa* of levels of clonal reproduction similar to those of a few other island species for which such information is available, while lower levels of clonality have been reported for endangered, mainland species, implies that clonality might play a more important ecological and evolutionary role in rare insular than mainland species. Even though there is a shortage of studies on the extent of clonal reproduction specifically on islands, it is reasonable to propose that clonal growth may offer an advantage especially in small and isolated populations, where clones may have a greater ability to persist than sexually reproducing individuals (Silvertown 2008). High rates of clonal propagation were actually found in mainland populations that, similar to those of island endemics, were small and marginal (i.e., rare or endangered species, populations of alien plants, or at the edges of species' geographic range; Silvertown 2008).

The occurrence of genetically identical individuals in all *R. microcarpa* populations results in a reduction in the already small population size of these populations. This further complicates the conservation status of this species, especially if considering that the high number of clonal individuals detected in *R. microcarpa* populations (74% of the sampled plants shared the same MLG with other samples) may represent an underestimation of the real incidence of clonality in this species, for adjacent plants (which might represent ramets of the same genet) were avoided during sampling. Notably, our results also showed that spatial distances among *R. microcarpa* individuals do not necessarily reflect the degree of genetic relatedness among individuals, highlighting the importance of molecular techniques in assessing the genetic characteristics and spatial distribution of individuals in populations thought to be affected by clonal propagation.

### Genetic differentiation among populations

The results show *R. microcarpa* to be genetically structured with high differentiation among populations (*F*_ST_ = 0.446). This finding is expected for island species with highly fragmented distribution (Frankham 1997; Carrol and Fox 2008) and is congruent with results of genetic analyses in other Canarian endemics. Francisco-Ortega et al. (2000) reviewed the genetic diversity of 69 species endemic to the Canary Islands and concluded that most of the genetic variation was explained by differences between populations.

The presence of private alleles in all populations, the high values of *F*_ST_ (Table 4) and the low migration rate indicate that populations of *R. microcarpa* are genetically isolated. Since isolation by distance was not detected, other factors affecting gene flow are more likely to explain genetic isolation than geographic distance. The two most differentiated populations (MUL1 and MUL2, *F*_ST_ = 0.920; Table 4), in fact, are spatially very close to each other, with only a road separating them. This suggests a lack on dispersal ability for *R. microcarpa* and highlights the susceptibility of this species to habitat fragmentation. Several factors could explain the low dispersal ability of *R. microcarpa*. For example, its seeds do not show any characteristics typical of a high ability to disperse (i.e., they are not fleshy and have no wings). Lizards, which are thought to be responsible for seed dispersal, are short-range vectors. Furthermore, since allelopathy is suspected to occur in some *R. microcarpa* populations (R. M. Coelo, pers. comm.), individuals that disperse to a different population might not necessarily be able to establish. Therefore, the presence and intensity of allelopathy could further reduce the already low migration rate among populations.

### Conservation implications

This study provided important insights into the genetic structure of *R. microcarpa* and demonstrated the high susceptibility of this species to extinction. The very small effective population size, low genetic diversity, and low levels of gene flow put at severe risk the persistence *R. microcarpa* and highlight the immediate necessity of measures for conservation. In situ conservation is essential and should aim to preserve as many individuals as possible, including the ones belonging to very small populations, since they can harbor unique genotypes. Concentrating conservation efforts only on the few, large populations or only on part of the populations, in fact, would result in the likely loss of genetic and genotypic variability for the species.

The main threats to *R. microcarpa* are habitat fragmentation, grazing, and competition with introduced exotic plants (i.e., *Opuntia maxima;* Bañares et al. 2004; Moreno 2008). Accordingly, in situ conservation should include agricultural and grazing control, in addition to measures to reduce introgression of alien plants. Ex situ conservation in seed orchards is also advisable, for the eventual reintroduction of seedlings belonging to the same population should restore genetic diversity and sustain fitness (Wilkinson 2001). However, this measure would only be successful if seedling establishment is not prohibited by allelopathy (R. M. Coelo, pers. comm.). Further research on the reproductive biology, dispersal ability, the presence of allelopathy, and its influence on seedling establishment is fundamental for planning more specific, potentially successful long-term conservation programs.

## Conclusions

To our knowledge, this study represents one of the few analyses of the effects of vegetative propagation on the genetic structure of endangered species on islands. We found that clonality positively affects the genetic diversity of the critically endangered endemic *R. microcarpa* by increasing allelic diversity, polymorphism, and heterozygosity. Even though clonality has mating costs related to inbreeding depression and pollen discount (Honnay and Jacquemyn 2008; Vallejo-Marín et al. 2010), our results indicate that clonal propagation may benefit endangered species. However, the increase in genetic diversity associated with clonal growth is accompanied by a progressive reduction in genotypic diversity, which is expected to ultimately lead to monoclonal populations (Balloux et al. 2003; Honnay and Bossuyt 2005). For this reason, the advantage of clonal growth may be only short-lived. As also suggested by Silvertown (2008), sexual reproduction might be indispensable to the long-term success of a species and clonal growth may play an important role in prolonging the time to extinction when sex is reduced or absent.

Our analyses revealed very low genetic variability for *R. microcarpa*. This result, together with the drastic reduction in genetic population size due to the detection of clonal propagation, makes the already critical conservation status of this endangered species even more problematic. Conservation management should aim to conserve as many individuals as possible, including those belonging to very small populations, for they can harbor very different genotypes that would otherwise be lost. In order to effectively manage and conserve populations of *R. microcarpa*, further research is needed regarding its reproductive biology, dispersal abilities, the presence of allelopathy and its influence on seedling establishment.

## Data Accessibility

Microsatellite genotype data used in this study are provided as Table S2, which are available in the online version of this article.

## Supporting Information

**Table S1.** Features of the 9 microsatellite markers used in this study. Shown for each marker are annealing temperature (T_a_; ^o^C), fluorescent label attached to reverse end of primer, size of the fragment (bp) and number of detected alleles (N_A_).

**Table S2.** Multilocus genotypes (MLG) based on eight polymorphic microsatellite loci across 73 *R. microcarpa* individuals. In the last column individuals sharing the same MLG belong to the same group. For abbreviations of populations and subpopulations see Table 1.
