# Supplementary material for: Effects of clonality on the genetic variability of rare, insular species: the case of Ruta microcarpa from the Canary Islands
Source: Ecol Evol. 2013 Apr 22;3(6):1569–79. doi: 10.1002/ece3.571 (PMC3686192; doi:10.1002/ece3.571)
Supplement: Supplementary file 1 [file ece30003-1569-SD1.doc]

Table S1 Features of the 9 microsatellite markers used in this study. Shown for each marker are annealing temperature (T_a_; ^o^C), fluorescent label attached to reverse end of primer, size of the fragment (bp) and number of detected alleles (N_A_).

| Locus | Ta | Label | Size | N_A_ |
| --- | --- | --- | --- | --- |
| RO57 | 55 | VIC | 210-230 | 5 |
| RO59 | 55 | FAM | 180-200 | 5 |
| RO62 | 55 | FAM | 160-210 | 7 |
| RO66 | 54 | NED | 190-200 | 4 |
| RO70 | 55 | VIC | 160-210 | 9 |
| RO71 | 55 | NED | 150-170 | 10 |
| RO72 | 55 | PET | 245 | 1 |
| RO77 | 55 | PET | 180-190 | 3 |
| RO79 | 55 | FAM | 230-270 | 8 |

Table S2 Multilocus genotypes (MLG) based on eight polymorphic microsatellite loci across 73 *R. microcarpa* individuals. In the last column individuals sharing the same MLG belong to the same group. For abbreviations of populations and subpopulations see Table1.

| Population | Individual | MLG (RO57/RO59/RO62/RO66/RO70/RO71/RO72/RO77/RO79) | MLG group |
| --- | --- | --- | --- |
| ALO | 1181  1182  1183  1184  1185  1186  1187  1188  1189  1190  1191  1192  1193  1194  1195  1196  1197  1198 | 226226/191197/158168/196198/198202/151153/181183/267267  226226/191197/158168/196198/198202/155157/181183/267267  226226/191197/158168/196198/198202/000000/181183/267271  226226/191197/158168/196198/198202/151153/181183/267271  226226/191197/158168/198200/198202/155157/181183/267267  226226/191197/158168/198200/198202/155157/181183/267267  226226/191197/158168/198200/198202/155157/181183/267267  226226/191197/158168/198200/198202/155157/181183/267267  226226/191197/158168/198200/198204/155157/181183/267267  226226/191197/158168/198200/198202/155157/181183/259267  226226/191197/158168/198200/198202/155157/181183/267267  226226/191197/158168/198200/198202/155157/181183/267267  226226/191197/158168/198200/198202/155157/181183/267271  226226/191197/158168/198200/198202/155157/181183/267267  226226/191197/158168/198200/198202/155157/181183/267267  226226/191197/158168/198200/198204/155157/181183/267267  226226/191197/158168/198200/198202/155157/181183/267267  226226/191197/158168/198200/198204/155157/181183/267267 | I  II  III  III  IV  IV  IV  IV  V  VI  IV  IV  IV  IV  IV  V  IV  V |
| MUL1 | 1199  1200  1201  1202  1203  1204  1205  1206  1207  1208 | 215226/187187/206206/194196/160160/161168/181181/237247  215226/187187/206206/194196/160160/161168/183183/237247  215226/187187/206206/194196/160160/161168/183183/237247  215226/187187/206206/194196/160160/161168/183183/237247  215226/187187/206206/194196/160160/161168/183183/237247  215226/187187/206206/194196/160160/161168/183183/237247  215226/187187/206206/194196/160160/161168/183183/237247  215226/187187/206206/194196/160160/161168/183183/237247  215226/187187/206206/194196/160160/161168/183183/237247  215226/187187/206206/194196/160160/161168/183183/237247 | I  I  I  I  I  I  II  II  II  II |
| MUL2 | 1209  1210  1211  1212  1213  1214  1215  1216  1217  1218  1219  1220  1221  1222  1223 | 228228/183191/168168/198198/166166/157161/183183/239239  228228/183191/168168/198198/166166/157161/183183/239239  228228/183191/168168/198198/166166/157161/183183/239239  220228/183187/168170/194198/166176/155157/183183/239253  228228/183191/168168/198198/166166/157161/183183/239239  228228/183187/170170/198198/166178/157161/183183/239239  228228/183191/168168/198198/166166/157161/183183/239239  220228/183187/168170/194198/166176/155157/183183/239253  220228/183187/168170/194198/166176/155157/183183/239253  220228/183187/168170/194198/166176/155157/183183/239253  228228/183191/168168/198198/166166/157161/183183/239239  228228/183191/168168/198198/166166/157161/183183/239239  228228/183191/168168/198198/166166/157161/183183/239239  228228/183191/168168/198198/166166/157161/183183/239239  228228/183191/168168/198198/166166/157161/183183/239239 | I  I  I  II  I  III  I  II  II  II  I  I  I  I  I |
| RC1 | 2232  2233  2234  2235  2236  2237  2238  2239  2240  2241  2242 | 213220/183183/170184/194200/160176/155159/181181/247267  213220/183183/170184/194200/160176/155159/181181/247267  213220/183183/170184/194200/160176/155159/181181/247267  213220/183183/170184/194200/160176/155159/181181/247267  213220/183183/170184/194200/160176/155159/181181/247267  213220/183183/170184/194200/160176/155159/181181/247267  213220/183183/170184/194200/160176/155159/181181/247267  213220/183183/170184/194200/160176/155159/181181/247267  213220/183183/170184/194200/160176/155159/181181/247267  213220/183183/170184/194200/160176/155159/181181/247267  213220/183183/170184/194200/160176/155159/181181/247267 | I  I  I  I  I  I  I  I  I  I  I |
| RC2 | 2243  2244  2245  2246  2247  2248  2249  2250  2251  2252  2253  2254  2255  2256  2257 | 220226/183183/172186/196200/160176/155159/181183/239247  220226/183183/172186/196200/160176/155159/181183/239247  220226/183183/172186/196200/160176/155159/181183/239247  220226/183183/172186/196200/160176/155159/181183/239247  220226/183183/172186/196200/160176/155159/181183/239247  220226/183183/172186/196200/160176/155159/181183/239247  220226/183183/172186/196200/160176/155159/181183/239247  220226/183183/172186/196200/160176/155159/181183/239247  220226/183183/172184/196200/160176/155159/181183/239247  220226/183183/172184/196200/160176/155159/181183/239247  220226/183183/172184/196200/160176/155159/181183/239247  220226/183183/172184/196200/160176/155159/181183/239247  220226/183183/172184/196200/160176/155159/181183/239247  220226/183183/172184/196200/160176/155159/181183/239247  220226/183183/172184/196200/160176/155159/181183/239247 | I  I  I  I  I  I  I  I  I  I  I  I  I  I  I |
| CED | 2258  2259  2260  2261 | 215228/187187/170170/198198/166200/164167/183185/237239  215228/187187/170170/198198/166200/164167/183185/237239  215228/187187/170170/198198/166200/164167/183185/237239  215215/187201/170170/198198/166180/164170/183185/237255 | I  I  I  II |
